# "The Hospital" Nursing Mirror

**Published:** 1899-10-14

**Authors:** 


"The Hospital" Nursing Mirror.

Being the Nursing Section of "The Hospital."

[Contributions for this Section of "The Hospital" should be addressed to the Editor, The Hospital, 28 & 29, Southampton Street, Strand, London, W.C., and should have the word "Nursing" plainly written in left-hand top corner of the envelope.]

### Notes on News from the Nursing World.

### THE NURSES AT THE FRONT.

Among the passengers by the special train to Southampton on Friday morning who attracted attention were the nurses who have been sent out by the Army Nursing Service to the base of operations in South Africa. Miss Ann Garriock, superintendent, has been in the service since May, 1886. She was trained at the London Hospital, where she afterwards was staff nurse for two years. From 1887 to 1891 she was at Malta Hospital, and from 1891 to 1896 at the Station Hospital, Devonport, her last appointment being at the Station Hospital, Woolwich, where she was sister, and in July she became superintendent. Miss A. C. L. Anderson has been in the Service since September, 1894. She was trained at the Children's Hospital, Nottingham, and also at St. Bartholomew's, where she was subsequently staff nurse. Since then she has been sister at Netley for most of her time. Miss A. A. Murphy, who has been in the Service at the Royal Infirmary, Phœnix Park, Dublin, since 1894, was trained at the Adelaide Hospital, Dublin, where she was afterwards staff nurse. Miss H. L. Neale, too, joined in the same year, having trained at the General Hospital, Birmingham, where she became successively assistant nurse and staff nurse. Miss Neale was sister at Netley from 1894 to June, 1897, and was then transferred to the Military Hospital, Canterbury. The other sisters have been several years in the Service.

### THE NURSES' QUARTERS IN THE HOSPITAL SHIP "SPARTAN."

Three nurses went out on Tuesday to South Africa in the s.s. "Spartan," belonging to the Union Steamship Company (Limited), namely, Miss Makepeace, Miss Tripp, and Miss Saunder. The "Spartan," which is one of the first vessels ever fitted out as a hospital ship, was prepared for her new duties by the company under the supervision of Major Wodehouse, R.A.M.C., who is in medical charge, and of Captain Herries, R.N. Prominent on her white sides is the huge Red Cross, emblematical of her mission. There are four sick wards in all—one aft for 26 cots, one on the starboard side of the saloon for 50 officers, and two others forward with 15 and 19 cots respectively. These cots are swung on short uprights fixed to the deck, and hang about a foot above it; and they are so arranged as to swing with the movements of the ship, or they can be fixed so as to keep quite steady. The cabins of the nurses are on the port side, just aft of the saloon. One large cabin is for the superintendent, and one is shared by the other two. These cabins are charmingly fitted up with cretonne hangings, chests of drawers, and writing-tables, and look the very picture of comfort. Specially-constructed lifts are arranged over the wards, so as to render the movement of the wounded from one bed to another as much as possible unnecessary. Refrigerating apparatus to produce ice on board at the rate of 10 cwt. a day is provided. There is a padded room for violent mental cases, and a large laundry and drying-room on deck. The operating-room is on deck, and is fitted with every latest improvement, including a complete installation of the Röntgen ray apparatus. Nearly 50 orderlies belonging to the R.A.M.C. sail in the ship to look after the patients, though we hope that their duties may be light. The "Spartan" is, of course, fitted with the electric light throughout.

### THE COLONIAL NURSING ASSOCIATION AND SOUTH AFRICA.

In the event of the War Office running short of nurses for service in South Africa application for help would probably be made to the Colonial Nursing Association. At present, however, the association has not been asked to send out any nurses to the seat of the war, and it is not expected that the request will be preferred. The last nurse who went to South Africa under the auspices of the Colonial Nursing Association was Miss Atkinson, who left England in April for private nursing in East Griqualand.

### ROYAL BRITISH NURSES' ASSOCIATION.

There are three meetings of the Royal British Nurses' Association imminent. The first chiefly concerns candidates for registration. The secretary hopes that all names will be sent in without delay, as the Registration Board reassembles, after an interval of three months, on Friday this week. On Friday, October 20th, the Executive Committee will make final arrangements as to the social and other engagements for the approaching winter session. The official announcements will appear in the November issue of the *Nurses' Journal*. On Friday, October 27th, a General Council will be held at five o'clock at 11, Chandos Street. It is not certain whether Princess Christian will have returned to town in time to attend, but it is hoped that she will, as the presence of the Royal President in the chair is always appreciated by the members of the Association.

### THE COUNTESS OF DERBY AT THE WHISTON INFIRMARY.

Last Friday Lady Derby paid a visit to the new infirmary at Whiston. She was received by the Rev. S. A. K. Sylvester, chairman of the Prescot Board of Guardians, who introduced her to Miss Parrish, the lady superintendent of nurses, and to Miss Grimshaw, the home sister. Having inspected the Nurses' Home, and expressed her pleasure at the arrangements for the comfort of the nurses, Lady Derby was conducted through the hospital and dispensary. In the children's ward she gazed for a few moments with tender sympathy at two dying babies, and was much attracted by a sweet, pink-faced little child of three years, who had one arm bandaged to her side, and only one little hand available to play with her toys. In the maternity ward the young mothers with their tiny infants elicited expressions of interest and pity from the visitor. She talked to the patients in quite a homely way, especially to one old lady who had lived somewhere on the estate. Lady Derby told the old woman about her sons, to one of whom she had been obliged to say good-bye only that day. He had "gone to the war." There are few ladies, of either high or low degree, who, after such a trying ordeal, would have found comfort in bringing sympathy and brightness to the inmates of the wards of a workhouse hospital.

### A GRACEFUL COMPLIMENT TO SISTER CECILIA.

The Sisterhood of All Saints' Home, Margaret Street, who have had charge of the nursing of University College Hospital since 1862, having recently resigned, it was felt by the committee of the hospital that the very valuable services rendered by Sister Cecilia, who for 12 years had worked as a ward sister and for 17 years had occupied the responsible position of lady superintendent of nursing, should be recognised. Accordingly, in conjunction with the members of the medical and surgical staff, they assembled in the boardroom on Wednesday last week, and presented Sister Cecilia, as the representative of the All Saints community, with a copy of one of Raphael's "Madonna," to which was attached the following inscription: "The Sisters of All Saints undertook the nursing of University College Hospital in 1862, and for 37 years devoted themselves to this work of charity. In grateful remembrance of their long association with the sisterhood the committee and staff of the hospital present to Sister Cecilia, who has been sister superior for 17 years, this copy of a well-known picture by Raphael."

### THE NEW FEVER HOSPITAL AT GLASGOW.

Several of our readers have expressed a desire for information respecting the opening of the new Fever Hospital in Glasgow, and there is natural curiosity as to the nursing posts. We learn that the hospital will not be ready for occupation before midsummer next year, and that then the nursing staff from the branch hospital will be transferred to it.

### SOCIETY OF CHARTERED NURSES.

The Society of Chartered Nurses steadily grows in popularity. It is a source of satisfaction to the Committee of Management that the staff more than maintains its reputation for efficiency. Numbers of newly-qualified nurses would only too gladly put their names on the books, but unless they are thoroughly experienced in private as well as hospital work, it is useless for them to try and do so. Nursing in hospital, where the patient and nurse are both under strict discipline, needs to be supplemented by experience of private work, in which the nurse has to learn to adapt herself to circumstances, to waive non-essentials, and yet to be firm in carrying out essential rules. The Society of Chartered Nurses consider that much of their success is due to their insistence upon the members of their staff possessing this insight into the difficulties of private work. It enjoys frequent opportunities of sending nurses abroad, and its members are, in fact, scattered into the far corners of the earth. The dearth of nurses in some of the provinces and in the less popular branches of nursing contrasts with the overwhelming numbers of applications for posts in connection with the larger and best known associations of the metropolis; and nurses who find it difficult to obtain employment in London would do well to inquire into the conditions and remuneration of work in less favoured localities.

### A POINT NOT CLEARED UP.

It appears from a report in the *Irish Independent* that the Roscrea Poor-Law Guardians some weeks ago "unanimously elected to the position of nurse at the fever hospital Miss Elizabeth Bradley, of Dublin." In spite of this unanimity, however, the Roscrea Guardians do not seem to have scrutinised Miss Bradley's qualifications very closely, for, first of all, her statement that she was a Protestant escaped their notice. Upon subsequently discovering this fact they appealed to the Local Government Board to annul the appointment. The appointment has accordingly been annulled, but not because of the plea urged by the Guardians. The latter were directed to proceed to a new election, "on the ground that Miss Bradley, although a trained nurse, was not a medical and surgical nurse within the meaning of the new regulations." From this we gather that the Roscrea Guardians did not study the new regulations as they ought to have done—a precaution which would have saved them from raising the religious question at all. But we should like to know whether the Irish Local Government Board would have annulled the appointment merely because Miss Bradley is not a Roman Catholic.

### THE GREAT MALVERN DISTRICT NURSING ASSOCIATION.

In consequence of the dissatisfaction expressed by certain medical men with regard to the nursing under the management of the Great Malvern District Association, there has been a general meeting of the subscribers of that organisation, at which the most conflicting views were expressed. Colonel Heywood, referring to the charge of inefficiency on the part of the nurses, said that one of the inspectors paid periodical, but unexpected, visits without the slightest warning, carefully inspected the work of the nurses, and afterwards made a general report, and that these reports, during nine years, had been of a very satisfactory character. On the other hand, Dr. Grindrod, the chief spokesman of the adverse critics, insisted that the nursing had not been by any means so satisfactory as it might have been, and urged that there was room for improvement on the part of those employed by the association. In particular, he maintained that the sanction of the doctor should in each case be obtained before sending out a nurse. Colonel Heywood, however, replied that "there were endless trivial cases among the poor in which the experience and training of the nurse were sufficient, without the necessity of calling in a medical man at all," and ultimately it was decided to form a duly elected committee, though this, apparently, neither satisfied Colonel Heywood nor Dr. Grindrod.

### A LESSON FOR THE WEST HAM GUARDIANS.

The "humour" of Poor Law Guardians, referred to in our issue of September 30th, has had its reward. One of the nurses appointed by the West Ham Board, after they had freely indulged in personal remarks about the candidates, has cancelled her engagement. In a letter announcing her intention, she said that "it would be better for her not to join a board which thought more of her complexion than of her capabilities."

### A "NURSE SUNDAY" AT ALFRETON.

At the annual meeting of the subscribers to the Alfreton Nurses' Fund last week, which happily combined business and pleasure, mention was made of a new departure in the year. For the first time a "Nurse Sunday" was observed in the town, and the innovation was so much appreciated that henceforth it is to be an annual fixture. Henceforth the offertories in all places of worship on the third Sunday in February will be given to the nurse fund. In spite, however, of this welcome support, the funds are lower than they have ever been before, and the committee appeal in particular to those who still give nothing to assist the work. The fact that the nurse paid 3,564 visits in the twelve months shows that her services are greatly needed,

### "A THEATRE NEXT DOOR TO A HOSPITAL."

It will be recollected that, commenting in June on the proposal to erect a theatre and opera house on a site immediately adjoining the North-Western Fever Hospital, we said: "If the noise of the performance is likely to interfere with the patients, retarding their progress, in spite of the most careful nursing, the London County Council will be justified in refusing consent to the plans for the new building." Apparently, the London County Council are satisfied that this result would follow the erection of the theatre, for they have declined to sanction the plans, and the project thus falls to the ground. In future, it is unlikely that a site next to a large hospital, however desirable it may seem, will be chosen for such a purpose.

### IMPROVEMENTS AT THE LOCK HOSPITAL.

Far-reaching alterations are in progress at the London Lock Hospital, which will affect both the staff and the patients. Last year the Prince of Wales' Hospital Fund made a grant of £500 on condition that the defective drainage was put in order. Opportunity was taken of the partial closing of the wards to make several structural improvements. The nurses' bedrooms are also being remodelled, but it will be some little time before the work is done and their occupants are in residence. During the holidays the matron, unfortunately, died suddenly. A fully-trained certificated nurse has been appointed to succeed her, and will enter upon her duties about the middle of November. The nursing staff, six in number at the present time, have charge of 120 patients when all the beds are in use. The work is naturally of a trying character; but happily it is supplemented by a rescue home. All patients, therefore, have the chance of returning to a happier mode of life, if they will. Of the 662 entering the hospital last year, 56 passed into the home, and were provided with situations; whilst a fair proportion were drafted into other homes of a similar nature. The management of the rescue work is distinct from the hospital branch, but the matron of the nurses necessarily has a position of peculiar responsibility.

### NURSING IN VIENNA.

Some severe and well-deserved strictures are made by a writer in the October number of the *Nineteenth Century* on the condition of nursing in Vienna. Despite of progress, despite of modern ideas of what is fitting, despite the prominent position of Viennese medical men in the scientific world, "the care of the sick," she says, "remains to-day at a point where England stood some fifty years ago." Here is a striking illustration in support of the assertion:—

"The nurses in the vast Allgemeines Krankenhaus, with few exceptions, are recruited from the lowest class of domestic servants, women who, because of incapacity, increasing years, or doubtful character find it difficult to get a situation elsewhere. Many look as if they had been accustomed to field work. They come in for a time to suit their own convenience, fulfil or neglect their duties as the case may be, and when anything better offers drift out again. Training, properly so called, they get little, except in the obstetric wards. Whatever they learn they pick up in haphazard fashion."

It is admitted that the pay is miserable, and that the conditions are unattractive. Otherwise, perhaps, it would not have been possible for Miss O'Conor Eccles to have described the following experience:—

"As we made our way to the hospital by the Alserstrasse gateway an elderly peasant woman, with weather-beaten face and gnarled fingers, passed us. On her back she carried a basket of wood. Her short skirt of yellowish-brown cotton stuff reached her ankles. Over it was worn a loose wrapper, or camisole, to match, girded in by apron strings round her thick waist. On her head was a little grey shawl. My companion indicated her: 'That,' he said, 'is one of the nurses.'"

Clearly, "Sairey Gamp" is alive and flourishing in Vienna.

### THE HOMES OF ST. BARNABAS.

Founded in 1895, and incorporated in 1899, the Homes of St. Barnabas may be described as a unique institution. Among the objects are, the provision of permanent homes for aged clergy who are past work, or who are incapacitated by incurable disease or other causes; and the provision of homes of rest and nursing homes to which sick clergy and those who have broken down in their work, and convalescents, can be admitted for longer or shorter periods. The Bishop of Rochester is president, and the Duke and Duchess of Somerset, the Hon. and Rev. Canon Littleton, the Hon. W. F. D. Smith, M.P., Mr. F. A. Blaydes, and Mr. C. O. Merritt Fox are among the members of the council. The honorary lady superintendent is Mrs. W. H. Cooper, and the nursing staff consists of Mrs. Churchill and Miss B. M. Rose, known respectively as Nurse Dorothy and Nurse May. The site for the first permanent home has been acquired near East Grinstead; and there is every reason to believe that the newly-formed Society for Homes for Aged and Disabled Clergy will obtain the measure of support it deserves.

### SHORT ITEMS.

Miss Rose Petty has been appointed a lecturer on "Health," under the London School Board. Trained at the London Hospital, Miss Petty was the first nurse appointed by the London School Nurses' Society. She lectured under the London School Board on "Home Nursing" last year, is the author of "Cleanliness in Children," and holds the certificate of the Sanitary Institute—of which she is an Associate—and First-Class in Advanced Hygiene, Science, and Art Department.—At the second annual meeting of the Cumberland Nursing Association, the Countess of Lonsdale in the chair, an alteration in the rules was adopted enabling more adequate remuneration to be given to the nurses. In future the rate will be from £85 to £100 per annum for Queen's nurses, and from £44 to £55 for cottage nurses.

### Lectures to Surgical Nurses.

By H. A. LATIMER, M.D. (Dunelm), M.R.C.S. of Eng., L.S.A. of London, Consulting Surgeon, Swansea Hospital; President of the Swansea Medical Society; Lecturer and Examiner of the St. John Ambulance Association, &c.

### RICKETS—NATURE OF THE DISEASE.

ALTHOUGH, strictly speaking, rickets is not a surgical disease, I have deemed it to be advisable to include it amongst my lectures to you, as it often leads to deformities in the bones which necessitate operative measures being taken by surgeons. You cannot have failed, during your work here, to have met with many children who have been admitted into our hospital for either the medical or surgical treatment of this complaint, and you cannot fail to be interested by the remarks which I am about to make on its nature, causes, and treatment. Although writers have described cases of congenital rickets —that is, of rickets existing at the time of birth of a child —it is exceedingly rare for this disease to show itself before the age of three months, and its most frequent time of appearance is at the period of weaning, when a child's food is being changed in character. The disease may appear at a later time, it is true, but in the majority of cases it shows itself between the ninth and fourteenth months. There is good reason for this peculiarity. It is at this age that milk, which has been—or should have been—the food by which the child has been nourished, is put aside in favour of a farinaceous diet; and as this time of life is one at which the digestive organs are peculiarly sensitive and are liable to become disordered in action, and as farinaceous foods, which are largely composed of starch, require complex acts on the part of various glands which manufacture secretions for the especial purpose of converting starch into sugar, without which conversion this body is unassimilable, unless a child is in good health, the new tax which is being levied upon his resources proves too much for his strength, and he becomes "out of health." From these remarks you will see that rickets is a disease which calls for the physician's help in the first instance; and you will also gather that if the little subject of it has been born of unhealthy parents, or has been brought up under unhealthy surroundings of any sort, it is peculiarly liable to supervene when any call is being made upon the child's strength of constitution. It is so far the result of faulty parentage and of unhealthy environment that, given the one or the other, it is pretty sure to appear on the scene in due course of time. Thus it is that the offspring of parents living lives of impoverished health in large cities, where they are deprived of fresh air, sunlight, and a suitable dietary during infancy, are peculiarly prone to show early signs of this complaint; and if we would successfully combat it we should take measures at an early stage in life for preventing it by improving the dwellings of the poor, preventing the overcrowding of their houses, and by diffusing amongst them a knowledge of the laws of health, and more rational ideas on the subject of the appropriate foods for young children.

Given, then, these causes of rickets, let us see how this disease shows itself, and what treatment it calls for at our hands. Sir William Jenner has drawn attention to three especial symptoms which mark it at an early stage. These are profuse sweatings, especially about the head and neck; restlessness at night, and a habit of kicking off the bed-clothes, and so lying in an uncovered state; and evident pain when handled in the nurse's arms. The two first of these symptoms are due to debility; the third is the result of a tender and sensitive state, in which pain results from pressure upon, or stretching of, delicate structures in the limbs. With these symptoms will be noticed a pale, flabby condition of the body generally; a disordered appetite and much thirst; an unhealthy condition of the bowels, in which either constipation or diarrhœa prevails, or, as is often the case, an alternating condition of the one or other disturbance in regularity; and an alteration in the shape and size of the bones, which is the occasion for the surgeon's help if he should be called upon the scene. The observer is not to be misled by an appearance of fatness in the child. Mere fat is no sign of health, but rather the contrary. If on examining a limb it is found that its plumpness is due to health, on rolling the flesh between the thumb and fingers it will be found to yield a substantial sensation of fulness and firmness; if, on the contrary, the child is "out of condition," the same test will show the apparent fatness to be unsubstantial, and the flesh will be flabby and of no true firmness. Flabby fat of this sort is a sign of ill-health, and marks an arrest midway in the processes of change in the body which should take place when life is being pursued in a natural manner. Under ordinary conditions our bodies are being continually wasted and repaired, old flesh undergoing fatty changes which fit it for being removed from the system, while the cells of which it is composed are replaced by new tissue; under certain conditions of ill-health these processes are not carried out in their entirety, and the fatty, flabby tissues which are formed as the semi-results of normal waste are retained in the system, to its detriment. But it is especially in the bones that the changes take place which mark this disease. They become soft and swollen, and very characteristic deformities in them ensue. The swellings are particularly noticeable at their ends where, by their apposition to other bones, they enter into the formation of joints. Thus it results that the wrists, knees, and ankles stand out very prominently, and, in severe instances of the complaint, there is a swelling of the heads of the ribs at their junction with the breast-bone, which is so peculiar that it has received the name of "the Rachitic Rosary."

Besides these swellings of the articular ends of the bones, everywhere in the system they are interfered with and affected by a softening process which is due principally to an absorption of, or want of proper deposition in them of, the earthy salts by which they altered from their originally plastic condition as membranes and cartilages into their rigid and unbending state as true bones. You can easily imagine what deformities in the body will result from such a weakening in the osseous system as this is. The softened shafts of the long bones will bend to the weight put upon them, or will become distorted by being dragged upon, here and there, by the various muscles which are attached to them; and the bones of the skull and of the pelvis will yield and become displaced and distorted in such ways that in after life the results of this disease during childhood are apt to be of the greatest seriousness. Thus it results, in the case of women, that the rickets of childhood may be a serious bar to child-bearing in adult life; for the pelvic bones may have been so altered in shape that the passages for the child during birth may be either so narrowed that it is impossible for it to be born at full time, or, if not so gravely disturbed as this, they may prevent a natural delivery taking place, and will necessitate the use of instruments during the labour. For in order that the "mechanism of labour" may proceed with safety to the mother and child concerned, it is essential that the bones of that part shall be of a certain shape, and placed at certain distances from each other, so that they may both allow of the passage of the child and direct the same in certain planes—as they are called—of the inlet and outlet of that cavity.

### Papers for Probationers.

### TAKING TEMPERATURES.

By a Sister.

There are two ways of taking a temperature, as there are of doing most other things—a right and a wrong way, or an intelligent and a mechanical.

To accomplish the latter put a thermometer under a patient's arm, or let him do so himself; after a minute or two tell him to hand it back, glance at the index, "chart" the result, whether 95 deg. or 108 deg., and go and do likewise by the next patient and the next, till you have "done all the temperatures." The results of this method can be attained with still less trouble and about as much accuracy by leaving the thermometers in their cases and marking "dots" in various places on the charts. This you will not need to be told is the wrong way of doing things, and yet I have frequently seen it in practice.

Now let me show you the right and intelligent way. There are five different parts of the body in which the temperature may be taken, namely, the axilla, groin, mouth, rectum, and vagina. The two last are the least often used, and the temperature of those parts would be a degree, and in the mouth about six points, or half a degree higher than in the axilla or groin. The axilla is the most often used for the routine-taking of temperatures, being the most convenient in the majority of cases. First see that the part is dry and free from perspiration, then shake down the index of the thermometer below 95 deg. and place the bulb under the arm in such a way that it is completely surrounded by flesh and not tucked in between the shirt sleeve, vest, or bandages, and the skin. If the patient has been lying with his arms above his head they must be put down to his side till the axilla, chilled by exposure, has regained its normal heat before the thermometer is inserted. When a patient is very thin the temperature should not be taken in the axilla, as it is almost impossible for the bulb to be in contact all round with the skin; it will rest in a hollow cavity instead, and the true body heat will not be recorded.

When the temperature is taken in the mouth the bulb must be placed under the root of the tongue, the patient being directed to close his lips on the stem but not his teeth, and must keep them closed till the instrument is removed. The mouth is impracticable where the patient is very ill or unconscious, as it is often impossible to get him to keep his mouth closed long enough, or to remain still enough for a true record to be obtained. Of course you would not attempt to put a thermometer into the mouth of a delirious or insane patient. Be extremely careful to clean and carbolise the thermometer thoroughly both before and after using it in the mouth.

The groin with children, and often in the case of unconscious adults, is the best place to take the temperature. The bulb of the thermometer should be placed in the groin, the stem being upwards, and the leg on that side crossed over the other to hold the thermometer firmly imbedded.

The rectum and vagina. Should you be told to take the temperature in either, direct the patient to lie on the left side with the knees drawn up, and after oiling the thermometer pass it gently till the bulb is quite covered. Do not forget to put screens round the bed nor to thoroughly clean the instrument.

So much for the various localities. The Sister of the ward will tell you in which of the five she wishes the temperatures taken, and you must take them nowhere else without her permission. When a temperature, usually taken in the axilla or groin, is taken in the mouth, rectum, or vagina, a note should be made of the fact, otherwise the apparent rise will be misleading. You will see the importance of placing the thermometer yourself, and yourself taking it out. If you let the patient put it in you do not know if it has been correctly placed, and if you let him take it out you cannot tell if it has remained where you put it.

Remember that there are differently-made thermometers, taking respectively half-a-minute, one, three, or five minutes, to register the heat of the body. Find out how long yours require, and be sure to leave them in the full time.

The best way to ensure cleanliness and to avoid breakage is to carry the thermometers in a small glass bowl containing carbolic lotion 1·20, and having at the bottom a piece of cotton wool, upon which the bulbs can rest as they stand upright in the bowl. On removing a thermometer from a patient, put it in the bowl and wipe it dry before placing it with the next case.

Now, as to where intelligence and honesty come in. Perhaps when you go to remove the thermometer, you will find that the patient has let it slip from its position, or has dropped it altogether. Then, if you are honest you will not think of the five precious minutes lost and hurry on to the next case, but you will remember that you are entrusted with an important duty, and you will shake down the index and replace the thermometer, holding it in place if necessary, to ensure an accurate record. Perhaps a patient's body feels cold, and yet the thermometer registers 103 deg. F., or higher; in that case you will take the temperature again with another thermometer, and report the matter to Sister. The instrument may be faulty and have "run up"; the patient may have tampered with it, and a second trial will show if the recorded temperature is the true one. Again, the mercury may stand at 98 deg, F., yet the patient may look flushed, seem hot, and complain of feeling chilly. An unexpected rise or fall requires corroboration, perhaps a thermometer under each arm, or in the rectum and axilla simultaneously. It is not sufficient to report merely a high or low temperature, but the fact of a rise or fall. The normal or usual temperature of health is 98·4 deg. Fahr., but with some people it is lower, sometimes 97 deg. Fahr. only; sometimes higher, 99 deg. Fahr. being normal. You will see, therefore, that if the thermometer registers 99 deg. Fahr. with a patient whose usual temperature is 97 deg. Fahr., it indicates a rise of two degrees, and means the same thing to him as a temperature of 100·4 deg. Fahr. does to a normal temperature of 98·4 deg. Fahr. After death from some diseases, the temperature rises to a great height—108 or 112 deg. Fahr. In some wards it is always taken after death, as a matter of routine.

Before leaving the subject of temperatures I must say a word or two with regard to rigors. A rigor is a shivering attack, accompanied by a sense of chilliness (in severe cases the patient's lips, ears, finger-tips are blue, or cyanosed) and a rise of temperature. The shivering may last from a minute or two to half an hour. A rigor is an important symptom, due to some interference with the heat-regulating centres in the brain. Rigors occur at the onset of many diseases, in septic and in malarial conditions, and frequently after catheterisation. The patient's temperature should be taken at the onset of the rigor, again in half an hour, and then in an hour's time. Always report a rigor at once to Sister, or anything which seems to you to resemble one.

Temperature is said to be :—

| | | | |
|---|---|---|---|
| Collapse ... ... ... | below | 97 | deg. Fahr. |
| Sub-normal ... ... ... | ,, | 98·4 | ,, |
| Normal ... ... ... | at | 98·4 | ,, |
| Pyrexia ... ... ... | above | 100 | ,, |
| Hyperpyrexia ... ... | ,, | 105 | ,, |

The height of the temperature in relation to pulse and respiration is very important to observe.

In conclusion, let me give you six golden rules for the taking of temperatures:—

1. Always shake down the index of the thermometer below 95 deg. Fahr.

2. Always put it in and take it out yourself.

3. Leave it in the full time.

4. Report a rise or fall, as well as a high or low temperature.

5. Never "chart" a doubtful temperature till you have taken it a second time.

6. Observe strict cleanliness with regard to the thermometers, and keep a special one for a septic or infectious case.

### Across the Seas.

PRIVATE NURSING IN THE SOUTH SEA ISLANDS.

Assuming that most people know sugar plantations are prominent among the industries of these islands, and that they are managed by European overseers, it may interest our home nurses to have the experience of one who has worked amongst them. The Islands are many and very scattered, taking from two to five days to reach by water, exposed to a pitiless sun, and, after it has set, to the miseries of mosquitoes and sandflies, which take a special delight in extracting English blood, raising immense blisters, generally on the hands and face. The swelling is so great that it is sometimes impossible to see where the hands leave off and the arms begin, or if the little insects happen to catch the eyelids one eye has to do duty the rest of the day. They do not treat all Europeans so badly as they treated me, and they rarely trouble the natives. Flies are numerous, so that they might come under the heading of plague. A native is generally employed at meal times in whisking them off the food.

The house in which the European overseer, his wife, and family live is built of wood, bungalow style, usually surrounded with cane fields. On the outskirts of these, shed-like buildings are provided for the coolies, who with their wives and families do the work of the fields and the menial work of the house. In household duties it is one continual round of teaching; they will not remember. Cooking is their best point—that they do fairly well with a little supervision; cleaning they do not see the need of and would not waste any time over it unless closely followed; neatness is quite out of the question; and they have no idea of washing garments thoroughly. When one considers that the nursing is generally maternity work, and that the impossibility of trusting to the natives results in the nurse washing many things herself, it is almost needless to say that she must not think of nursing only.

The Europeans receive you most warmly in their homes, and their sympathy with you does much to make matters seem easier than they are.

A great disadvantage to the climate is the heavy rain, called by the very modest name of "tropical showers." We English have never seen a downpour at home which would give an accurate resemblance to a "shower" in the South Sea Islands. I remember once leaving one of my cases to get down to the river, where a boat was to take me to meet a steamer. My way was through the cane fields, the cane being scattered all about. A single line of rail was laid down, on which a waggon is run, and a trolley is kept for the Europeans' use when they wish to ride down to the mill. A wooden plank forms the seat, and there is another for the back. It was pushed along by a couple of coolies, and being only a single line every two hundred yards or so, when we met a waggon being loaded with cane, we had to dismount. Trolley and luggage were carried to the front of the waggon, then we remounted and went ahead until we came to the next, when we went through the same performance again. This happened four times before we reached the terminus, and had it been summer the experience might not have been unpleasant; but it was what they call the wet season, *i.e.*, frequent showers. The sun had been shining before I left the house; within the next ten minutes down came the rain, and, in spite of English serge waterproof cloak, serge dress, &c., I was drenched through to the skin, with my shoes soaking. In a very few minutes the sun was out again, and I was just as quickly and thoroughly dried. This occurred three times before we reached the boat. The distance, along a most beautiful river, thickly wooded on both sides with overhanging trees, is covered in about four hours from landing-stage to the mouth of the river, where you join the big steamer. We arrived about five p.m., expecting to see the steamer about six; but none came. We waited until sundown, but in vain. By that time we had lost the tide for returning by the river, so there was nothing to do but to anchor and wait until the returning tide next morning at five a.m. The coolies on board knew very little English, and I no more Hindustani. They gave me to understand that the drinking water was all gone, but they would land at some native huts at the mouth of the river, beautifully shaded and wooded with cocoa-nut trees, and see if they could get any. They brought back a can full, about the colour of lemonade, with numberless specks floating about to be seen with the naked eye. As there was no prospect of getting anything different before ten a.m. next morning, I had to make the best of it, but used it sparingly. In most islands the water is very good. Close by a cargo ship was loading and unloading. She could not take passengers, and was timed to leave at two a.m. About midnight the officials came on to our little boat, and brought me coffee and bread and butter before they left.

Whilst the ship remained I did not notice the loneliness, but when I saw it disappear as a speck on the water, the desolation seemed awful. I had for company only half-a-dozen coolies, and myriads of mosquitoes and sandflies, which would not let me stay on one spot for five minutes. It was, however, only the *feeling* of loneliness. No Europeans could have shown more thoughtfulness and consideration than those coolies under trying circumstances—they were so thoroughly refined in all their actions. We returned with the tide at five a.m., landing at nine a.m., returning again at eleven a.m., that being the tide. Still no steamer. Back again by the next tide to the landing jetty, where I was kindly accommodated for the night by European strangers. Then back again next morning, and so on, backwards and forwards, for three days before the steamer arrived. Five days later I landed at the Government town where my headquarters were situated. The experience, which certainly might have been filled with horrors, I remember with the greatest pleasure. When the cases happen to fall in the town the life is home-like, counteracting in a measure the loneliness of the smaller islands, but under all circumstances time seems to pass very rapidly. From a financial point of view the difference in salary between private nursing here and nursing at home is an increase of about £25. Deduct £10 for extras which you will want from home, and duty, and there is a margin of £15. If you are lucky enough to retain your health and forget the disagreeables to which you must submit there is plenty of happiness to be found in the fields of private nursing in the South Sea Islands.

### Nurses of To-Day.

### THE SYMPATHETIC NURSE.

She was one of the first nurses I encountered on my entrance into hospital life, and well I remember her. Above the medium height, with calm grey eyes, and fair hair worn turned back from her forehead and surmounted by the regulation cap, Nurse Helen was the very beau-ideal of a hospital nurse. She came forward to meet me, saying the matron had asked her to take charge of me and initiate me into my duties, and I, a forlorn girl leaving home for the first time, fell a victim to her charming sympathetic manner. Without any words being necessary, she at once divined my home-sickness, and set to work to try and comfort me. As we became better acquainted I found this part of comforter was her special *rôle* in life. Everyone came to her with their grievances, both small and large, and she seemed to find time for all, having

"A heart at leisure from itself,
To soothe and sympathise."

That was the secret of her popularity, I think, and it was that which made her such a good nurse. The heaven-born gift of sympathy which she possessed so largely, the power she had of mentally putting herself into the place of each one of the poor sufferers she tended, made her understand so easily the needs of each. And how they all loved her! As she passed down the ward dull eyes grew brighter at her approach, while weak voices said appealingly, "Stay and talk to me awhile, nurse," or "The pain is more bearable when you sit by me." And she would always spare a few minutes to rejoice with one over his prospect of soon returning home, or to cheer up another to whom the weary time of convalescence seemed long. To each she gave some of her large store of sympathy, thereby helping them to take a brighter outlook.

Once I heard the matron telling her she must learn to look on her patients more as "cases" and less as "individuals," or her strength would not stand the strain. But it was not in her nature to look on her work solely from a professional point of view. Her heart was too much in it for that.

I was fortunate in starting my new life in her ward, and can never forget what I owe to her teaching and example. Never did she lose her temper even with the most irritable and exacting of patients, and there was one who was enough to try the patience of a saint. He was an old man who was brought in late one Saturday night. It was a common case. A little too much to drink, and a slippery pavement after a slight fall of snow. Coming out of the public-house, Old Sam fell, and broke his leg. A naturally irritable temper was made still more so by the unaccustomed restraint, for Sam had never before spent a day in bed in his life, and had been able, even at seventy, to do his day's work with "the best on 'em." Nothing pleased him. The soup was sure to be too hot or too cold, his bandages were too tight, he wanted his "bit o' baccy," and so on. But Nurse Helen at last acquired some influence over him, and he was once heard to say, "That fair-haired wench ain't a bad lass arter all." A high word of praise from Old Sam.

I ventured once to ask something of her life before she came to the hospital, and learnt that it had been full of trouble. She had passed through the furnace of affliction, which, instead of hardening her, as alas! it does so many, had destroyed the dross of her character, leaving only the pure gold.

Nurse Helen has left us now, and taken up mission work in London, and much do we miss her. There amongst the poor of an East-end parish she finds plenty of scope for her sympathies. She has known suffering and sorrow herself, and day after day labours to diminish them for others, finding her reward in the love and gratitude of the poor outcasts her care has benefited.

### Appointments.

Victoria Hospital, Folkestone.—Miss S. M. Armitage has been appointed Matron. She was trained at the Children's Hospital, Pendlebury, and King's College Hospital, London. Her subsequent appointments have been sister for one year at Birmingham Infirmary, sister for two years at Lewisham Infirmary, and sister for two years at the Hospital for Sick Children, Great Ormond Street.

Peterborough Infirmary and Dispensary.—Miss Alice King has been appointed Matron. She was trained at St. Thomas's Hospital, and has since been seven and a half years staff nurse at St. Thomas's, sister, night superintendent, and ultimately the assistant matron of the Great Northern Central Hospital.

Hove Sanatorium.—Miss Florence Hill has been appointed Matron. She was trained at King's College Hospital, and was afterwards for five years charge nurse at the Jamsetjee Jeejeebhoy Hospital, Bombay. For the last three and a half years she has been sister of the ward for abdominal operations at the Sussex County Hospital.

Borough of Folkestone Sanatorium.—Miss Lucy A. M. Hughes has been appointed Nurse-Matron. She was trained at Chelsea Infirmary. Subsequently she has been charge nurse at the Western Fever Hospital, Fulham, and for six and a half years superintendent of nurses.

Royal Isle of Wight Infirmary and County Hospital, Ryde.—Miss Georgina H. Sked has been appointed Matron. She was trained at the Royal Infirmary, Liverpool, and has since been assistant matron at the Royal Albert Edward Infirmary, Wigan.

Chester Isolation Hospital.—Miss Marion Bland has been appointed Matron. She was trained at Chester General Infirmary, and subsequently became a member of the private nursing staff.

### Minor Appointments.

South Shields Workhouse Infirmary.—Miss E. Howe has been appointed Head Night Nurse. She was trained by the Glasgow Sick Poor and Private Nursing Association; was two years previously at the City of London Infirmary, Bow; and has since been three years charge nurse at the Union Infirmary, South Shields. She has recently obtained the L.O.S. certificate.

Park Fever Hospital.—Miss Anna Riley and Miss Kate H. Todd have been appointed Charge Nurses. Miss Riley was trained at Shoreham Infirmary, and she has subsequently been charge nurse at the Birkenhead Infirmary, and staff nurse at the Infirmary, St. George's-in-the-East. Miss Todd was trained at the Royal Infirmary, Bristol, and has since been engaged in private nursing.

Taunton and Somerset Hospital.—Miss Beatrice Horswell has been appointed Sister. She was trained at Salisbury Infirmary, and was subsequently charge nurse of the male medical and accident wards, Salisbury Infirmary; sister of male wards, and also out-patient and casualty departments, Suffolk General Hospital.

The Infirmary, East Dulwich Grove.—Miss Josephine Bourdillon has been appointed Sister. She was trained at the London Hospital, and has since been temporarily assistant matron at the British Hospital, Endell Street. She holds the L.O.S. certificate. Miss Alice Mary Jarratt has also been appointed Sister. She was trained at St. Saviour's Infirmary.

Homes of St. Barnabas, Incorporated, for Poor, Aged, and Disabled Home and Missionary Clergy.—Miss Catherine H. Francis has been appointed Charge Nurse. She was trained at St. Lucy's Home, Gloucester, and has since been assistant and charge nurse at the Ely Union Infirmary.

Burnley District Sanatorium.—Miss Lucy Trew has been appointed Charge Nurse. She was trained at the Monsall Hospital, Manchester, and has since been charge nurse at Sunderland and nurse-matron at the Isolation Hospital, Hinckley.

Lexden and Winstree Union Workhouse, Stanway.—Miss Agnes M. O'Malley has been appointed Superintendent Nurse. She was trained at Brownlow Hill Infirmary, Liverpool, and has since been engaged in private nursing.

### A Book and its Story.

MR. HAWTREY'S HUMOUR.

No one, surely, can take Mr. G. Hawtrey's delightful absurdity seriously, and yet so cleverly is it conceived that there may be readers, lacking in the sense of humour, who will read it with perfect gravity and probably pronounce it to be an exceedingly foolish book. Certainly "Caramella"* is not written for the Plain Person, who prefers a nice straightforward story, free from that fine vein of satire which dominates the conception of this apparently simple, if somewhat allegorical, romance. The Island of Caramella, the scene of much amusing topsy-turvey-dom in military, diplomatic, political, and domestic departments, does not strike us as being a thousand miles, perhaps not a hundred yards even, from our own shores. The delightful condition of do-nothing-ness into which everyone drifts is only an extreme view of what would happen in our own country if the theories of some sections of the community were reduced to practice. Disarmament, peace at any price; office hours from twelve till four; a fortnightly bank holiday; woman controlling, or trying to control, important official departments (man having become an effete creature and a person of no authority), is a not impossible vision of the future to those holding extreme views on this interesting question.

Jack Fanshawe is the idle son of an idle father, who, like other parents in a similar dilemma, finds the development of this failing particularly irritating to contemplate in one to whom he is specially devoted. At Eton Jack had been a favourite with everyone; he thought, after leaving, of going into the army, but was advised by his genial tutor to do nothing so foolish, as he would certainly fail in his examination. "This, however, he did not do. He never went in for the examination at all, for the crammer said that it would be perfectly ridiculous for him to make the attempt. So Jack went home and had a talk with his father." "What is to be the next move? I haven't the least idea," said Jack. "My farm bailiff," said Mr. Fanshawe, "tells me that he is looking out for a ploughman." Jack laughed; he knew his father's moods, and he didn't mind subtleties of this kind. "You laugh," said Mr. Fanshawe. "Can you suggest anything better? I can't." The stage did not appeal to Mr. Fanshawe, nor the Church to Jack, as examinations are necessary for that career. Business was the only alternative. After two months in the office of a flourishing hop merchant in the Borough Jack was dismissed. The head clerk in the office found his authority undermined; the office was scarcely better than a "bear garden" in consequence of the little diversions which Jack indulged in when the back of the dignified Jeffreys was turned. "Jack was adored by the other clerks." A second conversation took place at Fanshawe Court, with the result that Jack was despatched to the Cape with two hundred pounds to start with, and on the way meets with Ajax, a young Caramese gentleman, who invites him to Caramella. "It's ever so much pleasanter than Johannesburg." "But should I get on there? I've got to sink or swim; and I don't want to sink if I can help it." "You won't sink in Caramella, my friend. Give it a trial." "Is Caramella so very delightful?" "Ah!" sighed Ajax. It would be difficult to convey an idea of the intense meaning expressed in that long-drawn "Ah!" "How about the language?" "It is a very easy one. I'll teach you." "That's awfully good of you. But you mustn't be hard on me for my mistakes in grammar." Ajax smiled. "There are no mistakes in Caramese grammar. Whatever you say is correct." "It seems to me that is the language I have been pining for all my life. What a pity they didn't teach it at Eton." Jack goes to Caramella, and finds no difficulty with the language. "Sometimes the ladies with whom he was conversing did not understand what he meant. But they were never at a loss, under the circumstances. . . . It is unnecessary to point out the enormous advantages of this sort of conversation. Everyone knows there are times when it is so much better not to understand what people mean." The Caramella people were known by one name only. Ajax derived his Homeric title from ancestors supposed to have come over with Ulysses. "It must be a great satisfaction to belong to a really old family," observed Jack. "In Caramella," answered Ajax, "it is regarded as a matter of the highest importance. . . . The royal family were descended in a direct line from Ulysses himself, but that is now extinct." On their arrival in Caramella, Jack and his friend proceeded at once to the stately home of Helen and Bellerophon, the parents of Ajax. "His mother and sister bubbled over with pleasure at seeing him, but neither rose from the depths of the arm-chair, or the hammock, in which they were severally reclining." It was an unwritten law in Caramella that no one exerted themselves unnecessarily. "You can be just as fond of a person sitting down, or lying at full length, as you can when standing up." In this lotus land of beauty and delight, Jack speedily finds himself at home. He takes his first taste of the fruit himself from the hands of the fair Daphne, Helen's daughter, and, being a strong, muscular young Englishman, in spite of his latent indolence, he finds it has no enervating effect, only a soothing, restful one. That this condition was easily discarded is easy of belief when we are told that after a brief residence in the island lazy Jack Fanshawe finds himself President of Caramella! Here is a little summary of the existing conditions of the settlement: "At first sight the idea of Jack, a young man only just out of his teens, governing Caramella will seem to quiet, sensible people positively ridiculous. But you must try to realise how utterly different was the state of affairs in this quaint island to anything with which you are able to compare it. The system of government was not an ideal one; but it managed to do the work very well, and no one wanted to change it. The Caramese were glad to be governed, and were profoundly grateful to anyone who would take the trouble off their hands. It is true there was a Foreign Office, but it had no relations whatever with any other country. There was a Colonial Office, but Caramella had no colonies. There was an Admiralty, but there were no war ships and no sailors. The Home Secretary had nothing to do and he did it most conscientiously. . . . The army was a relic of the Monarchy. When the Government became a Republic there was some idea of disbanding the soldiers; but the notion was given up because the President thought he would enjoy the pomp and display produced by the colour of the uniforms on state occasions. To prevent the rifles going off unexpectedly the gun barrels were all made of wood. For the supply of swords and bayonets the Government made a special contract. The weapons were obtained from a country in Europe and were precisely similar to some which had been proved by actual experience to bend whenever used in action. How Jack conducts the affairs of State as President we must not disclose, nor how he combats the fair Armila, his predecessor's daughter, who, until his advent, had reigned supreme in her father's department. There was an unwritten law in Caramella that "if anything was to be done the women would have to do it." But Jack proves here, as elsewhere, that where an Englishman is at the head of affairs he does his duty. He fought valiantly and successfully till obliged to lay down the weapons of self-defence and own himself vanquished at last by the fascinations of Armila. There is an almost studied absence of style about "Caramella" which the author explains as being "my way of telling a story, which, if you do not approve, I cannot help it." However, it is a very diverting method, if it lays claim to no special literary style, and one that suits the humorous situations with which the book abounds.

* "Caramella." By G. D. Hawtrey. (Published by Arrowsmith. 6s.)

### How Nurse Lilian Spent Her Holiday.

ANSWERING AN ADVERTISEMENT.

It may interest some of my sister nurses to hear how very pleasantly and happily one of their number has spent her vacation. I cannot but recall with the greatest possible pleasure every moment of the "holiday" from the time I left Victoria until my return five weeks later, nor do I remember a single incident without realising that the factors which helped to make it so thoroughly enjoyable and home-like were the unfailing kindness, courtesy, and consideration of my delightful hostess and her friends, for whom I shall ever entertain the warmest gratitude.

After finishing my training for a "Special subject" under rather trying circumstances, I thought how nice it would be could I only get away from the broiling heat of town, to some cooler spot, where I could appreciate either the pure fresh country air, or a sea breeze wafted from the ocean. So—surely Providence guided my eyes—glancing carelessly over the advertisements in one of the leading papers, I read that a lady living on the south coast offered hospitality and travelling expenses to a gentlewoman (trained) for four or five weeks, in return for companionship and occasional light duties in nursing.

I grasped the fact that my application would only be one of possibly a hundred. Imagine then my surprise and pleasure upon receiving by return of post an answer requesting full particulars as to where I was trained, my social position, and credentials! These I soon supplied, and within the week I was engaged to take care of a lady who wished for "congenial companionship combined with the confidence a nurse naturally inspires, should she require her services." Of course, my thoughts and ideas concerning my surroundings for the next few days occupied my mind fully, and I started, I will confess, with just a wee bit of trepidation, which was almost overpowered by the anticipation of a long holiday by the sea. I engaged my seat by placing my dressing-bag and papers in the compartment of a train which seemed almost overcrowded on a Saturday in August, making use of the few minutes before it started to take in a supply of what fresh air there might be about by walking up and down the platform. Mine was not one of the four coveted corner seats, but the only middle one vacant in the carriage. But that one "middle seat" promised a little bit of excitement before we left Victoria. My attention was arrested by hearing some one saying "nurse" once or twice, and when I gathered that an angry argument was proceeding between two men, in the compartment where I had deposited my belongings, and that the train was just starting, I felt some hesitation in entering. However, in I got, to find an anæmic-looking man declaring he had as much right to that "middle seat" as any nurse, and he meant to have it or none at all; and a gentleman in the corner seat equally determined that he should not. I was in time to hear the latter say that "he had early learnt that civility and respect were a nurse's due, and that in his presence they should always have it," adding, "and any man laying claim to be a gentleman, as you do, would not have to be asked to give way to a lady, let alone a nurse." My heart warmed to this noble tribute to our work and uniform, and after our objectionable fellow traveller (promising to "see my champion again") had unwillingly vacated my "middle seat," I warmly thanked this gentleman in the name of all nurses. In recording this incident I must make mention of a previous courtesy I met with that day—an action as gentlemanly in its way as the one just told. After finding my box amongst the many piled up for that train on the entrance platform, I requested a porter, who, with pail and brush, was rapidly labelling luggage, "If he would be so kind as to put a label on my box for L—— ?" Without turning round he informed me he was not the labeller for L——. I must go to "that one over there," pointing with his brush. But, as I passed him to try and procure the service from "that one over there," he stopped me saying, "I didn't see, ma'am, as 'ow you were a nurse; course I'll label your box, and put it in the van, too. I've had no end of kindness from nurses, me and my missus has."

My holiday at the outset promised well, so I left London with a heart full of gratitude and expectancy. Without any more adventures I finally arrived at L——, only an hour late. After a short drive to a comfortable modern house near the sea, and changing my dusty cloak and bonnet for apron and cap, I descended to the drawing-room for a very welcome cup of tea, where I was introduced to my hostess by her fresh and buoyant young niece, who had shown me to my room. A pretty, comfortable, cosy drawing-room it was too, but my attention was rivetted upon the lady of the house, and my thoughts were, "Surely this will be but little holiday for me! I've a patient that will need nursing, and careful nursing too, to judge by her thin, pale face and form. Oh, how ill she looks!"

I truly hope, and I believe, from what she afterwards told me, that my expression was "professional," and not an index to my thoughts. Her greeting was most cordial, and she told me she was an invalid from shock following an operation, but that, as a rule, she was able to go about her home quietly and wait upon herself. She was unable to bear the fatigue of a railway journey this year, and, in order that her relatives might have no anxiety about her during their absence, she had consented to have "a trained nurse" with her until their return. I very soon grew accustomed to her fragile appearance, and found her bright and entertaining, full of the things and doings of the day. My room was exceedingly comfortable, facing due south, and each morning I felt more and more refreshed. After breakfast, and having given to my "patient" the very little attentions she required, I was free to spend my morning with a book on the sea front till lunch, or to amuse myself in any other "holiday" way I might see fit. In the afternoon it was just the same; I was to do exactly as I liked if I wished to give satisfaction. I really think that my only "work," if one could really call it so, was making tea in the afternoon, when my dear hostess would lie on her invalid couch and listen and talk to the many friends who came to see her each day; which, instead of tiring, seemed sometimes to rest her. After dinner, and I had seen her to bed, she would always beg me to go and listen to the band for an hour or so. Her main object seemed to be to give me as little to do as possible, and to procure me as much pleasure and enjoyment as lay in her power. I met many very nice people who often asked me out to tea, &c., and when her own relatives returned I remained another week to make their acquaintance. As I had not taken my machine with me, my kind friend placed her niece's bicycle at my disposal, and many little excursions to places of interest (particularly one to a perfect "nursery," cultivated and kept by an invalid clergyman) were thoroughly enjoyed and appreciated.

I often think as I look back on my holiday that I might truthfully call my late hostess an ideal invalid; carrying out in the events of her daily life the lessons learnt from the Master's example; neither preaching nor talking "cant," but just living calmly and peacefully; always bright and happy; full of loving sympathy. If only there were more like her, who, in the same spirit of thoughtful kindness, would give a rest and holiday to a nurse who, perhaps, otherwise might have to go without one.

### Echoes from the Outside World.

AN OPEN LETTER TO A HOSPITAL NURSE.

THE seventy-fifth anniversary of President Kruger will be memorable for the fact that on that day the Boer Ultimatum rendering war between Great Britain and the Transvaal unavoidable reached London, astounding not only Ministers, but sympathisers with the Transvaal, by its impertinence. Not only was it required that British troops should not be allowed to assemble in the neighbourhood of the Transvaal, and that all our soldiers sent to South Africa since June 1st should be withdrawn, but we were calmly bidden to prevent any of those now on the high seas from landing at any of our own ports! This last demand reveals the depths of the ignorance which prevails in the Republic, and the overweening arrogance of its Government. I agree with Lord James of Hereford that "women hate cowards"; and, as he suggested in his speech at Aberdeen on Tuesday evening, the Government would deserve "to be scouted from office as craven cowards" by men and women alike if they had listened for a single moment to such proposals. The temper of the country has, I think, been fairly reflected by the enthusiastic reception afforded to the New South Wales Lancers as they marched through the streets of the capital; by the satisfaction with which Sir Alfred Milner's proposal to the Lord Mayor to open a Mansion House Relief Fund on behalf of the refugees at Cape Town was hailed; and by the patriotic letter of Lord Rosebery, who characteristically declares that "a situation has been created beyond party polemics."

---

IT is strange that the week of the Church Congress, designed to promote Christian love and kindness, should have been the week in which the Boer Government deliberately decided to provoke war. On Monday afternoon, when, owing to the chivalry of the organisers, the ladies' meeting opened the proceedings, the Ultimatum had not reached England, so that the war-cloud did not hang over it, and the gathering was a grand success. The Albert Hall, with 8,000 or 9,000 people filling all its vast galleries and parterres, is always an imposing sight, and most of the listeners, thanks to the enormous sounding-board over the platform—which suggested a huge umbrella and looked far from beautiful—seemed to hear the speakers fairly well. The Bishop of London's little address was delightful. He wrapped up his advice in such a palatable shape that it will not be easily forgotten, and he appeared to hit just the right nail on the head. Wives are so painfully apt, when they have been married a few years, to speak of their husbands as a nuisance, quite forgetting, as the Bishop reminded them, that it was the wife who chose the husband. The clever way in which he suggested that if woman were really the superior person she believed herself to be it would be easy for her to take the first step towards preventing a quarrel, because she would do it so much more gracefully, was most diplomatic, and made me smile a big smile. Mrs. Creighton's story of the little boy and the fruit, though it caused amusement, brought the tears to many a mother's eyes. An early guest at a dinner-party, being an intimate friend of the family, asked leave to go and survey the arrangements of the table before the other guests arrived. The room was dimly lighted, and as she stood there she heard little feet patter down the stairs, and a child entered. Seeing no one he climbed a chair, took a large peach, and ran off. As the lady sadly considered whether she ought to tell the child's mother of the theft, the little feet returned. Mounting the chair once more, the peach was returned to the dish, and the boy exclaimed triumphantly, "Sold again, old devil," without noticing the lady in the shadow. If only ou children could thus be made strong to resist temptation, how the sorrow and sin of the world would be diminished!

---

THE greatest feature of this end of the century seems to be the marvellous strides made in telegraphy. We have scarcely recovered from the surprise caused by Signor Marconi's invention, when yet a greater discovery treads close upon its heels. As long ago as last May it was rumoured that a system of quick telegraphy had been invented in Hungary. Quite recently, demonstrations of the working of the new system have been given in the presence of official representatives of Hungary, Germany, and France, and the directors of the great American cable companies. (By the bye, why did not England send a representative?) The result seems to have been marvellous. It is stated that it took just nine seconds to transmit a message of 220 words from Budapest to Berlin, and when it is in working order the new apparatus will be able to send 100,000 words in an hour. The only apparent drawback is that the messages have to be previously prepared after they are handed in at the office, the signals being perforated upon endless paper slips in the Morse code. But as a great number of people can prepare these slips at the same time, the minutes required for preparation need not be many.

---

IT is said that all nations, as well as individuals, must either go forwards or backwards; there is no middle course possible. If this be the case, I fear that France has, for the nonce, adopted the retrograde movement. Following hard upon the court-martial at Rennes comes the introduction of the bull-fight into the capital, and the enjoyment of brutalising scenes by the Parisian world of fashion. Elegant women, beautifully dressed, accompanied by Frenchmen who appeared attired for "sport," took advantage of the special trains run on Sunday last to flock to Deuil in such large numbers that the circus was packed from top to bottom. There was no doubt as to the nature of the entertainment. It had been largely advertised, and great stress had been laid upon the fact that the bulls would be *killed!* Had not the first bull turned into the arena been naturally a peaceful animal, the probability is that the killing would have been on the other side, for a bull in a fury, rushing amongst a crowd of ladies, would have done some serious damage. As it is, I feel glad that after he jumped the palisade in order to escape his tormentors, he must at least have trampled upon a few of the sight seers beforehe was shot and stabbed. No defence of the entertainment can be made, that like cock-fighting and glove contests in England, it was held without the knowledge of the authorities, for the Prefect of the Department of the Seine and Oise was present in a conspicuous position. The English Press has been laudably outspoken in condemning this last amusement on the part of the French people, which will probably confirm the *Gaulois* in the opinion it expressed last week that "the English are the common enemies of Europe." This seems a little hard when, if it were not for British travellers, half the hotels on the Continent might put up their shutters!

---

SINCE writing about Spanish brandy I find that it can be procured at any of our large wine merchants. It is very unlike French cognac in flavour, though about the same colour. I made a point of tasting it, and, had I not been told the contrary, I should have thought it a new liqueur with a suspicion of sherry about it. It is not nearly so fiery as the brandy we are accustomed to resort to in illness, and is also much more moderate in cost.

### Everybody's Opinion.

[Correspondence on all subjects is invited, but we cannot in any way be responsible for the opinions expressed by our correspondents. No communication can be entertained if the name and address of the correspondent is not given, as a guarantee of good faith but not necessarily for publication, or unless one side of the paper only is written on.]

### A MATRON'S DUTY IN REFERENCE TO RULES.

"To Follow Right" writes: In answer to "A Late Lady Superintendent," may I say that surely no matron ought to "allow things to go on which she knows to be wrong." If the committee and the matron differ as to rightness of rules and discipline, would not the wisest plan be for the matron tactfully to point out to the committee in what way the rules could be amended or made more workable? Or, perhaps a tactful appeal to the medical staff for help might be of use. But under no circumstances can it ever be right to allow wrong things to go on. For a woman to damage her own reputation as matron is a small part of the mischief she does; she harms the morals of herself and her subordinates, and she lowers the tone of the hospital. Depend upon it,

"Because Right is Right, to follow Right
Were wisdom in the scorn of consequence."

### NURSING IN FEVER HOSPITALS.

"Matron" writes: I am surprised at the experiences described in your columns. Surely no hospital, fever or general, can do its duty properly to the patients without a permanent staff of efficient head nurses, and these cannot be obtained if they have not regular probationers under them, bound for a certain time. We have a three years' course here, and I find our medical superintendent most particular that no nurse shall be made an assistant till she is properly qualified by experience in all wards. We might easily fill our vacancies by taking nurses who are anxious for six months' experience for their own benefit. We do take a certain amount of these, when we have enough work, but only as probationers, our primary object being to train our own nurses well, and we do not give the charge of wards to any except those of our own training. We take them back as head nurses after their general training, or send them for a year's surgical and gynæcological work. We have a very nice class of nurses, and more applicants than we can take. No nurse can realise till she undertakes fever work what valuable experience is to be met with—quite different to any she may obtain in a general hospital.

### SLIPSHOD WAYS.

"One Who Looks On" writes: I am much struck by observing into what casual and slipshod ways nurses seem to drop after they have been doing private nursing for a while, and I should like to know why this is, and whether others have noticed the same thing? I have repeatedly seen nurses—who in hospital were smart, clean, thorough in their work—lapse after a spell of private nursing into anything but smart ways! A kind of casualness seems to come over all their work. Things are not done up to time; glasses, cups, &c., are left to lie about dirty either inside or outside the patient's room; there is a general sloppiness in all the work, and an inclination to potter vaguely throughout the day, with a distinct tendency towards messiness and want of method. It seems a great pity that nurses should let themselves fall into ways of this sort, for it is no more difficult in a private house than in a hospital to keep to regular hours, to do your work up to time, to put everything away in the place where you took it from when you have finished with it, and to keep your patient's room in immaculate order. Few things are more annoying for a sick person than to be "pottered" round, and to have things done in a muddle. It seems a pity that nurses do not carry their hospital discipline with them in all their after work.

### THE BABY'S CORD.

"Perplexed" writes: I should be so glad if some of my fellow-readers of the "Mirror," who have had long practical experience in monthly nursing, would kindly tell me which way they have found the best to dress the cord. Almost every book on the subject has a different method, and these are not always clearly explained. In my own training I was told to have two small pads of absorbent cotton, to make a hole in one through which the cord is passed, turned up, and the other pad placed over it and the binder secured, the dressing not being touched until the cord drops. I bathe the child on my knees, change the binder daily and see that no swelling or redness appears round the cord. Occasionally this answers satisfactorily, the cord dropping about the fourth day, leaving a dry, flat navel; but more often it keeps on until the seventh or eighth day, and then the navel is not always as flat as I should like it, although subsequent pressure improves it. At other times there is a moisture about the navel which necessitates dressing with boracic powder for some time after. I also feel afraid that the hard, dry cord may irritate the infant during the last two or three days. On the other hand, it seems to me that if I were to change the dressing daily, it would be necessary to thoroughly moisten it with warm water in order to remove it without pulling on the cord—and would not this keep the cord soft and thus retard the process of drying and dropping? I am very anxious to know the best way, so I hope you will forgive me taking up space in your columns.

### Presentation.

Woolwich Union.—Last Friday evening Miss A. L. McKie (who has just resigned the position of matron of the infirmary, which she held for twenty years) was presented by the chairman of the Guardians, on behalf of the Board, with the following resolution, beautifully illuminated and framed: "Testimonial. To Miss Annie Louisa McKie, Matron, Woolwich Union Infirmary. The Guardians of the Woolwich Union desire to express their deep regret that it is necessary for you to resign your position as matron, which you have held so worthily for twenty years. They have pleasure in placing on record their sense of the high standard of integrity you have maintained, and especially that you have never allowed the trying character of the work to dull your Christian sympathy with the afflicted and helpless. The Guardians wish you many years happy life in your well-earned rest.—Signed on behalf of the Board, W. G. Dawson (Chairman of the Board), W. I. Squires (late chairman of the Board), J. Harper (late chairman of Infirmary Committee), C. E. Escreet (chairman of Infirmary Committee). September 28th, 1899." A very handsome silver toilet service, to which many of the Guardians and the whole of the staff contributed, was also presented to Miss McKie at the same time, and after several complimentary speeches had been made the recipient gracefully acknowledged the gifts.

### Novelties for Nurses.

### THE COMBINED BELT AND CORSET.

It has been well said that many of the objections to corsets arise from the fact that figures are laced in to fit the corsets, and not the corsets cut to fit the figures. The makers of the "Domen" Belt Corset have taken the greatest care to study feminine health and comfort. The belt has been largely advertised and is widely recommended by medical men. Now that it has been combined with a well-cut corset it will probably become more popular than ever, because the same support is maintained with a diminution of bulk and weight. The corset is cut in accordance with anatomical requirements; freedom of chest expansion is secured by a narrow insertion of elastic down each side under the arms; whilst firm support where needed is guaranteed by the addition of the belt. Ladies who have much walking or standing find it a preventative against fatigue, whilst as a surgical appliance it relieves much pain and discomfort. The prices compare favourably with those of any good corsetières. They range from 17s. 6d. to 25s. The corsets wear well; it is estimated that they will last a year without needing repairs, and at the end of that time if well mended they can be worn for another year. They are stocked in all sizes both for long or short waists, whilst a pair can be made to measure at a trifling extra cost. The company's head depôt is at 456, Strand, W.C.

### For Reading to the Sick.

"THOU shalt love the Lord thy God with all thy heart and all thy soul."

The time is short—
Therefore, with all thy might,
Labour for God and right;
Pause not for heats and shadows of the day,
Fail not for difficulties of the way.
Be true, be pure, be strong!
Eternity is long.

The time is short—
Sin, misery, and despair
Darken the earth and air;
Therefore do thou with heaven intercede,
And for thy brethren, ere they perish, plead.
Pray for the prayerless throng!
Eternity is long.

The time is short—
Therefore, my brother, love,
Love always! God above
Is one with thee in this. O, take
His crown of thorns, and thine own self forsake.
Love, spite of pain and wrong—
Eternity is long. —*Shirley Wynne.*

CONSCIENCE.

To love God really is to refrain scrupulously from whatever His love forbids, and to do all it enjoins; it implies continual self-renunciation, a continual war upon our earthly passions; it means humbling one's spirit, crucifying one's flesh, with all its lusts and evil longings; it means resisting the world's delusions, the stream of this world's ways and customs, the allurements of evil example. In short, it means a will steadfastly set to please God in all things, a resolution to displease Him in nothing. Is not such love in very truth a light of the conscience before which no dark corner can remain uncleansed? And how else, save by such diligent searching love casting its penetrating light in everywhere, and helping us to drag out every adverse thought or habit, can we hope to attain to that perfect Love which is "the fulfilling of the law"?

Guided by Love, then, let us each look carefully upon our own conscience, and see how far we are loving our dear Lord with all our heart, all our mind, all our soul, and all our strength, not only in avoiding great and obvious sin, but in the diligent cultivation of all those virtues and graces which are as precious flowers in the Lord's garden, pleasant and acceptable in their fragrance to the Master of the vineyard. —*Sidney Lear.*

Softly on the silent night
Falleth from God,
On weary wanderers
Over life's road;
And as the stars on high
Light up the darkening sky,
Lord, unto Thee we cry
Father above! —*Blatchford.*

### To Nurses.

IN order to increase and vary the interest in the *Mirror*, we invite contributions from any of our readers in the form of either an article, a paragraph, or information, and will pay a minimum of 5s. for each contribution. All rejected manuscripts are returned in due course, and all payments for manuscripts used are made at the beginning of each quarter, *i.e.*, January 1st, April 1st, July 1st, and October 1st.

### Notes and Queries.

The contents of the Editor's Letter-box have now reached such unwieldy proportions that it has become necessary to establish a hard and fast rule regarding Answers to Correspondents. In future, all questions requiring replies will continue to be answered in this column without any fee. If an answer is required by letter, a fee of half-a-crown must be enclosed with the note containing the enquiry. We are always pleased to help our numerous correspondents to the fullest extent, and we can trust them to sympathise in the overwhelming amount of writing which makes the new rules a necessity.

Every communication must be accompanied by the writer's name and address, otherwise it will receive no attention.

*Cripples.*

(19) I should be glad to know if there is an institute where a poor helpless cripple woman could be received without payment or at a small charge?—*M. T.*

(20) Can anyone tell me if there is any home where a young man with a broken back can be admitted? He fell from a cart of hay two months ago. He could pay about 10s. a week.—*Matron.*

There are a number of charities and homes, but it is impossible to name them all, or to select such as are suitable to individual cases. We must, therefore, refer our querists to Burdett's "Hospitals and Charities," a copy of which can be obtained from the Scientific Press for 5s., or it can be seen at THE HOSPITAL Office.

*Books.*

(21) I shall shortly establish a private nursing home in this district, and should be glad to know if there are any books published giving information concerning the management of same. The work would principally consist of maternity cases.—*L. O. S.*

We have no knowledge of any books on this subject. Of course, there are many books on midwifery, but we presume that you are already a qualified midwife.

*Nursery Lotion.*

(22) "A District Nurse," whose former communication appears to have been overlooked, kindly writes: I have found in district work that "Creolin"—one or two teaspoonfuls to the pint of water—is an invaluable lotion for cleansing the heads and hair of school children. It is a good and safe preparation to use. I have not known it to fail. Carbolic soap may be used with the creolin, or an ordinary soap if preferred.

*Hastings Hospital.*

(23) Will you kindly inform me if the Hastings Hospital is a recognised training school for nurses? It contains 74 beds.—*Nurse I.*

You should write to the Local Government Board, Whitehall, for information. It is hardly probable that they will recognise the training in so small a school as sufficient qualification for the post of superintendent nurse.

*Journal of Mental Science.*

(24) When and where is the *Journal of Mental Science* published, and what is the price?—*S. Y.*

Messrs. J. and A. Churchill, 7, Great Marlborough Street, W., publish this journal quarterly in January, April, July, and October. Price 5s.

*Vaporiser.*

(25) I have one of Dr. Woodward's (of Chicago) hot-air, nickel-plated vaporisers for nose or throat; height 15 in., weight 7 lb. It has been used one month. Can you tell me the best way of disposing of it, by sale?—*M. J. H.*

Apply to the English agents, or, if worth while, advertise it.

*Probationer at 33.*

(26) Will you kindly tell me if it is possible to become a probationer at a hospital or infirmary at the age of 33, or is it only by paying a premium that one can do so? I am trained in monthly nursing.—*A Constant Reader.*

See the list of provincial hospitals of over 100 beds in "The Nursing Profession: How and Where to Train." There are several given that receive suitable candidates up to 35 years of age.

*A General Hospital.*

(27) What constitutes a general hospital?—*Ignoramus.*

A hospital which, in contradistinction to a hospital for special diseases or a special class of patients, admits men, women, and children, whether medical or surgical cases.

*Smoky Chimneys.*

(28) Is there any cure for a chimney that always smokes. The sweep has been, and found no soot to speak of, and a cowl has been put on, but it is no better. It is a short chimney, facing east. Can anything more be done to prevent it smoking so badly?—*A. G. W.*

We hardly believe in a chimney that *always* smokes. Did the sweep pass his brush right out at the top?

*Holiday Home.*

(29) Will you please give me the address of the Paris Holiday Home for Nurses, also the terms? I am going to Paris in a few weeks' time, and should be glad to stay there.—*A. C.*

The London headquarters for the Paris and Continental Association—the home and office of the secretary—is 45, Eastbourne Terrace, Hyde Park, London. W. She can give information of all British and American benevolent and residential institutions for women workers in Paris.

*Domestic Economy.*

(30) I shall be much obliged if you will give me the address of any of the regular schools of domestic economy where a lady may learn all that pertains to housekeeping, in or near London.—*P. B.*

You can learn everything that pertains to housekeeping at the Polytechnic Schools. Address Regent Street, W.

